# Developing and Implementing a Training Programme for Medical Students to Become Peer Educators in Simulation-Based Education

**DOI:** 10.1007/s40670-024-02058-0

**Published:** 2024-05-07

**Authors:** Adam F. Roche, Olivia Jagiella-Lodise, Rebecca Kirrane, Claire M. Condron

**Affiliations:** RCSI SIM Centre for Simulation Education & Research, RCSI University of Medicine and Health Sciences, 26 York Street, Dublin 2, D02 P796 Ireland

**Keywords:** Learning, Innovation, Simulation, Pedagogy

## Abstract

Peer assisted learning is a useful strategy for medical students to learn from one another in a safe, structured capacity. As a pilot, we designed a training programme in collaboration with medical students to equip them with the knowledge, skills and abilities to act effectively as peer educators in simulation-based education.

**Supplementary Information:**

The online version contains supplementary material available at 10.1007/s40670-024-02058-0.

Peer-assisted learning (PAL) in simulation-based education (SBE) is a learning environment where students assume the role of educator and teach their peers [1]. Advantages of this approach include decreased dependability on experienced educators to teach, and it provides students with reassurance that trained student simulation educators (SSEs) are delivering teaching ethically and efficaciously in a safe learning environment.

High learning standards in SBE are influenced by creating a psychologically safe learning environment where students can feel emotionally capable of learning without concerns about facing consequences for making mistakes [2]. Those providing feedback in SBE who have received little or no training may compromise this, thus hindering the learners’ capacity to effectively learn. Therefore, without this vital training and support for peer feedback, anticipated gains are unlikely to occur.

As a pilot, we aimed to design and implement a training programme to recruit SSEs and equip them with the knowledge, skills and abilities (KSA) to act effectively in an informal basis as peer educators in SBE at the Royal College of Surgeons Ireland (RCSI), University of Medicine and Health Sciences.

The author team (AR, RK, CC) established an SSE committee, and four medical students (OJ-L) joined this committee as a result of a recruitment drive afterwards. The ethos behind this mutual partnership between staff and students was of shared decision-making, in order to promote authentic student engagement. This ethos was also intended to act as a teaching tool for SSE’s, to maintain equal peer relationships and impede the emergence of any potential hierarchy in student led training sessions that would follow.

After questions from an established PAL planning and implementation framework [3] were evaluated during initiation, themes for training development emerged (Appendix 1 in the Supplementary information). The committee expanded on these themes and described the training programme’s five phases in greater detail (Table 1). The programme completion timeline was 6 months from inception.

**Table 1:** Summary of each phase of peer simulation educator training programme

**Phase**	**Module**	**Title**		**Hours**
**1**		Introduction into simulation-based education (SBE)		3
**2**		Pedagogy – simulation educator training		
	**1**	*Topic: learning theory*		
		**Aims**	To inform the learner of the learning theories which underpin SBE	
		**Learning outcomes**	Understand the goals of the RCSI simulated teaching programme Understand the principles of experiential learning using SBE Be aware of learning theory that supports SBE	
		**Reflection**	“Learning theory and you”, 500 words	6
	**2**	*Topic: learning conversations*		
		**Aims**	To inform the learner of best practice from a number of perspectives including health and safety, psychological safety and the qualities of the facilitator	
		**Learning outcomes**	Demonstrate a critical awareness of the features and application of a safe learning environment and SBE methodologies Explain the philosophical underpinnings of experiential learning in SBE Demonstrate an effective prebrief Critically evaluate their own ability to create and maintain experiential learning in SBE	
		**Reflection**	“Creating a safe container”, 500 words	8
	**3**	*Topic: scenario development*		
		**Aims and outcomes**	To design an SBE scenario using best critical design practices	
		**Reflection**	Create your own acute care clinical simulation scenario, 650 words	4
	**4**	*Topic: reflective teaching and peer coaching*		
		**Aims**	To gain insight into the processes of examining experiences with the intention of learning or gaining new insights to improve your practice	
		**Learning outcomes**	To critically evaluate your own practices and beliefs when delivering SBE To understand the mechanisms associated with providing honest feedback to peers	
		**Reflection**	Reflective practice journal entry, 500 words	8
	**5**	*Topic: debriefing: “digging deeper”*		
		**Aims**	To provide clear structure for debriefing and facilitate the application of a framework for post event simulation debriefing	
		**Learning outcomes**	Discuss the rationale for debriefing in SBE Critical assess frameworks to structure debriefing Structure a debriefing in an organized fashion Demonstrate critical awareness of strategies to explore performance	8
**3**		Exposure to other simulation-based training programme		8
**4**		Prepare and pilot inaugural training programme		16
**5**		Committee reflection – training programme evaluation		2
**Total number of hours for programme completion**				**63**

As part of the introduction phase, SSEs attended an in-house workshop delivered by other committee members, aimed at giving SSE’s foundational knowledge of psychological safety in SBE, prebriefing and debriefing. Furthermore, it provided an opportunity for any questions and answers as well as additional discussion on the ethos and terms of reference of the training programme. Fulfilment of the pedagogical phase occurred when the SSEs completed phase two, which comprises a five module training course hosted on Moodle™. SSE’s were also recommended to write journal entries at key junctures throughout training, to aid reflective learning and programme evaluation at the end of training.

Given the SSEs affiliation with the emergency society (EMSoc) in RCSI, they were introduced to a postgraduate emergency medicine SBE training session as support faculty. Learners and faculty were made aware of the student’s participation and gave permission. The objective was to observe the practical application of the pedagogical components they had learned from phases one and two and provide mentored experiential training to our SSEs.

The PAL-SBE programme implementation in RCSI, which involved implementing previously developed simulation scenarios, was the next phase. SSEs would also take the lead in pre- and debriefing these scenarios as well as act as embedded participants. When needed, other committee members were present to provide support to SSE faculty. Following the pilot event, the committee met for a period of reflection to discuss the programmes effectiveness. Summative feedback from the learners with regard to the delivery of the simulation scenarios was very positive, with all participants agreeing that the SSEs facilitated the session in a clear, logical and concise manner. Additionally, all participants unanimously indicated that they had gained new skills in managing interactions with patients and colleagues. Participants also felt that the sessions “helped them to think outside the box, and also improve their communication skills”, and others felt they learned “more about the secondary survey approach in the management of the trauma patient”.

This approach to KSA assimilation was deemed reasonably achievable by all SSEs, and the content was deemed appropriate. In subsequent iterations of the SSE programme, attempts will be made to reduce the number of hours to complete the training, as SSEs highlighted moderate challenges in completing the training in its entirety within the requisite timeframe. Weekly SBE training sessions are scheduled by university societies; by reducing the need for experienced educators to lead on these sessions, the use of SSEs maximises the impact of these training sessions in a safe and productive manner. Although they are unpaid roles, SSEs who successfully completed all components of this programme are eligible for course completion certification.

### Supplementary Information

Below is the link to the electronic supplementary material.

Supplementary file1 (DOCX 21 KB)

## References

[1] Nunnink L, Thompson A, Alsaba N, Brazil V. Peer-assisted learning in simulation-based medical education: a mixed-methods exploratory study. BMJ Simul Technol Enhanc Learn. 2020. 10.1136/bmjstel-2020-000645. 10.1136/bmjstel-2020-000645 PMC8936843 35515740

[2] Turner S, Harder N. Psychological safe environment: a concept analysis. Clin Simul Nurs. 2018. 10.1016/j.ecns.2018.02.004. 10.1016/j.ecns.2018.02.004

[3] Ross MT, Cameron HS. Peer assisted learning: a planning and implementation framework: AMEE Guide no. 30. Medical Teacher. 2007. 10.1080/01421590701665886 10.1080/01421590701665886 17978966

