# Supplementary material for: Developing and Implementing a Training Programme for Medical Students to Become Peer Educators in Simulation-Based Education
Source: Med Sci Educ. 2024 May 7;34(4):743–5. doi: 10.1007/s40670-024-02058-0 (PMC11296977; doi:10.1007/s40670-024-02058-0)
Supplement: Supplementary file 1 — Supplementary file1 (DOCX 21 KB) [file 40670_2024_2058_MOESM1_ESM.docx]

Appendix 1 – PALS project framework responses

Background

Q1. What is the current situation and context in the curriculum?

**Currently, for informal teaching scenarios, students frequently give themselves peer feedback in simulation-based education (SBE). Often, these students have little or no experience or training as educators, which may compromise the psychological safety of learner groups.**

Q2. Why is this PAL project being considered now?

**Resulting from an impromptu conversations between RCSI SIM staff members and medical students enrolled in undergraduate programmes in RCSI, who expressed concerns with their peers' lack of training in the pedagogical aspects of teaching SBE.**

Q3. Who is responsible for the project and who will lead it?

**RCSI SIM staff members from the student simulation educators (SSE) committee- Adam F. Roche (AR), Claire M. Condron (CC), Rebecca Kirrane (RK), will be responsible for the committee. To promote a culture of collaborative decision-making, the initiative will be led by all committee members, including medical students.**

Aims

Q4. What are the aims and objectives of the project for tutors?

**To equip the tutors with the knowledge, skills and abilities to safely deliver SBE for peer learning in RCSI.**

Q5. What are the aims and objectives of the project for tutees?

**To enhance SBE learning opportunities and foster a peer-learning culture where tutees can feel free to make mistakes without fear of suffering unfavorable emotional repercussions.**

Q6. What are the aims and objectives of the project for the institution?

**The current RCSI strategy, which encourages authentic student engagement inside the faculties, benefits from this partnership between students and staff.**

Tutors

Q7. Who will be tutors and how will they be recruited?

**Students who are in their penultimate or final year of medical school will get an expression of interest form. Due to the fact that the students would have gained significant experience prior to these two senior years, they were taken into consideration for this pilot project. No specific knowledge or skills were deemed required for tutors to be deemed suitable applicants. A decision was made by AR, CC, and RK to include all students who expressed an interest and foster their talents, in this pilot programme.**

Q8. What training will tutors require and how will this be provided?

**It is believed that exposure to different SBE training programmes is particularly important since it gives students a practical perspective that comes from the pedagogical training phase. However, decisions on specific modules and subsequent criteria to be included in this programme will be made based on information elicited as part of this questionnaire and brainstorming from all finalised committee members.**

Q9. How else will tutors prepare themselves and reflect afterwards?

**Prior to rollout, sufficient and pertinent training will be given, and reflective learning journals should be kept throughout the project in order to properly reflect and evaluate at the conclusion.**

Tutees

Q10. Who will be tutees and how will they be recruited?

**Other medical students who engage in ad-hoc examination preparation, and form part of student society events.**

Q11. What related prior knowledge and experience will tutees have already?

**Knowledge and experience garnered as part of their medical degree programme.**

Q12. What information and preparation will tutees require before the interaction?

**Not required, as pertinent information to learning activities will be made explicit to them in the simulation event pre-brief. During this phase, tutees are given explicit guidelines for interaction, which is crucial for creating and maintaining a psychologically safe learning environment.**

Interaction

Q13. What will be the format of the interaction, and what resources are required?

**Resources such as manikins—both low-fidelity and high-fidelity—that satisfy the requirements for achieving learning objectives are necessary for SBE. The required teaching spaces that mimic the clinical arena are also required. Lastly, consumables required to execute clinical tasks, such as needles and cannula may also be required.**

Q14. What would be a typical plan of activities during the PAL interaction?

**Assuming the required student sim educator training has been completed, a typical PAL interaction follows the below format:**

- **Simulated scenario pre-brief (create rules of engagement and a psychologically safe learning environment)**
- **Execution of scenario (the task)**
- **Simulated scenario de-brief (cement learning objectives)**

Q15. When and where will PAL interactions occur, and how will they be arranged?

**This is all takes place in the RCSI SIM learning spaces. Students arrange and execute learning sessions themselves.**

Evaluation

Q16. What feedback will be collected from participants and how will it be used?

**Summative feedback in the form of post-event questionnaires will be sought from participants, in order to analyse learning sessions and make suggested improvements to future iterations.**

Q17. How else will the project be piloted and evaluated?

**An extra day of SBE activities will be scheduled at the International Conference for Healthcare and Medical Students (ICHAMS) at RCSI, which will be targeted as the pilot phase in this project. Following this, the training programme as a whole will be evaluated qualitatively by all students who took part, with reflective learning journals an integral part of this process. The following questions are proposed:**

- **What part(s) of this programme did you feel worked particularly well?**
- **What part(s) did you feel could be improved upon?**
- **Did you have sufficient time to complete the training?**
- **Do you have anything else to add?**

Q18. What are the academic hypotheses and how will they be tested?

**We believe that we will successfully equip SSE’s with the knowledge, skills and abilities to effectively lead on and deliver SBE to peers. Summative feedback from student peers and feedback from SSE’s at the end of the programme will aid the evaluation process.**

Institution

Q19. Who are potential stakeholders in the project?

**Staff from RCSI SIM, medical students, porters, security personnel.**

Q20. What are the staff time and funding implications of the project?

**Funding was not require for this project. There were no concerns in relation to staff time to dedicate to this project.**

Q21. How could the project be developed, and how might it affect the curriculum?

**This project will be developed collaboratively with all committee members. We don’t envisage that it will negatively impact the undergraduate curriculum, as it is supplemental learning only.**

Realisation

Q22. What are the potential pitfalls or barriers to the success of this project?

**Sickness or pressures associated with regular undergraduate studies that prevents student from completing the project in the given timeline. Mitigating circumstances will be taken into consideration, and allowances will be made for students to complete the project with an extended timeline. During the introduction phase, students will be reassured that their regular studies will take precedence over this project, and that realistic deadlines for completing each training component will be created in collaboration with each committee member.**

Q23. What are key points on the timeline for this project?

- **Recruitment**
- **Introduction and initiation**
- **Training**
- **Pilot**
- **Evaluation**

Q24. What actions need to be taken to develop the project, and by whom?

**Following the establishment of the SSE committee, any developments resulting from committee discussions will be deliberated upon and put into action.**
